# (*S*)-1,5-Dibenzyl-3-*tert*-butyl­imidazol­idin-4-one

**DOI:** 10.1107/S1600536808018461

**Published:** 2008-06-25

**Authors:** Jian-Feng Zheng, Jian-Nan Guo, Su-Yu Huang, Bo Teng, Li-Ren Jin

**Affiliations:** a Department of Chemistry, Key Laboratory for Chemical Biology of Fujian Province, College of Chemistry and Chemical Engineering, Xiamen University, Xiamen 361005, People’s Republic of China

## Abstract

The title compound, C_21_H_26_N_2_O, was obtained as an unexpected by-product when attempting to prepare (*S*)-2-benzyl-*N*-*tert*-butyl-1,2,3,4-tetra­hydro­isoquinoline-3-carboxamide from (*S*)-2-benzyl­amino-*N*-*tert*-butyl-3-phenyl­propanamide and dimethoxy­methane. The mol­ecules are linked by weak C—H⋯O hydrogen bonds, generating linear chains parallel to the *b* axis. C—H⋯π inter­actions provide further stability for the crystal structure. The planes of the two phenyl rings make a dihedral angle of 84.1 (1)°. The absolute configuration was known from the starting material.

## Related literature

For related literature, see: Allen *et al.* (1987 ▶); Pavel *et al.* (1993 ▶); Jin *et al.* 2005 ▶.
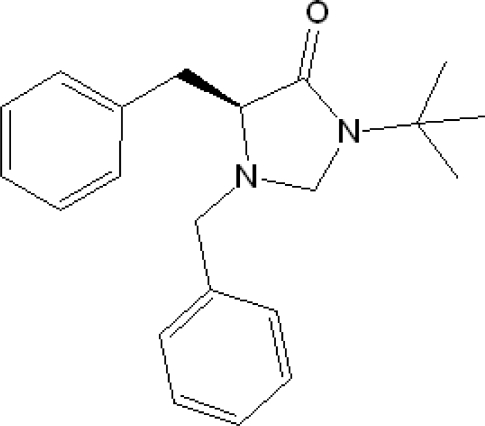


## Experimental

### 

#### Crystal data

C_21_H_26_N_2_O
*M* *_r_* = 322.44
Orthorhombic, 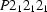

*a* = 9.4112 (6) Å
*b* = 11.4713 (7) Å
*c* = 17.0556 (11) Å
*V* = 1841.3 (2) Å^3^
*Z* = 4
Mo *K*α radiation
μ = 0.07 mm^−1^
*T* = 173 (2) K
0.62 × 0.45 × 0.23 mm

#### Data collection

Bruker APEX CCD diffractometer
Absorption correction: multi-scan (*SADABS*; Bruker, 2001 ▶) *T*_min_ = 0.957, *T*_max_ = 0.984
8034 measured reflections
2047 independent reflections
1824 reflections with *I* > 2σ(*I*)
*R*_int_ = 0.023

#### Refinement

*R*[*F* ^2^ > 2σ(*F* ^2^)] = 0.042
*wR*(*F* ^2^) = 0.107
*S* = 1.00
2047 reflections
217 parameters
H-atom parameters constrained
Δρ_max_ = 0.34 e Å^−3^
Δρ_min_ = −0.18 e Å^−3^

### 

Data collection: *SMART* (Bruker, 2001 ▶); cell refinement: *SAINT* (Bruker, 2001 ▶); data reduction: *SAINT*; program(s) used to solve structure: *SHELXS97* (Sheldrick, 2008 ▶); program(s) used to refine structure: *SHELXL97* (Sheldrick, 2008 ▶); molecular graphics: *ORTEPIII* (Farrugia, 1997 ▶); software used to prepare material for publication: *SHELXL97*.

## Supplementary Material

Crystal structure: contains datablocks I, global. DOI: 10.1107/S1600536808018461/bt2725sup1.cif

Structure factors: contains datablocks I. DOI: 10.1107/S1600536808018461/bt2725Isup2.hkl

Additional supplementary materials:  crystallographic information; 3D view; checkCIF report

## Figures and Tables

**Table 1:** Hydrogen-bond geometry (Å, °)

*D*—H⋯*A*	*D*—H	H⋯*A*	*D*⋯*A*	*D*—H⋯*A*
C6—H6*A*⋯O4^i^	0.99	2.48	3.439 (4)	164
C17—H17⋯*Cg*^ii^	0.95	2.68	3.621 (4)	169

Symmetry codes: (i) 
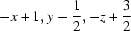
; (ii) 
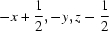
. *Cg* is the centroid of the C7–C12 phenyl ring.

**Fig. 1.**
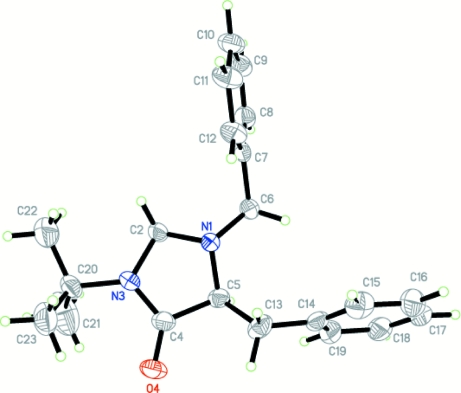
The molecular structure of (I) with the atom-labeling scheme, showing 50% probability displacement ellipsoids. H atoms are drawn as spheres of arbitrary radius.

**Fig. 2.**
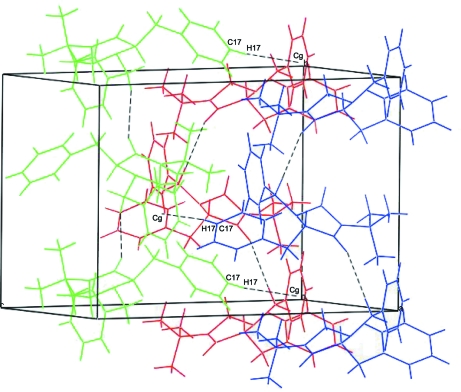
The packing of the molecules, viewed down the a axis. C—H···π and hydrogen bonds interactions are shown as dashed lines. Cg is the centroid of the C7 / C12 phenyl ring.

## supplementary crystallographic information

### Comment

In our studies on the synthesis of (*S*)—*N*-*tert*-butyl-tetrahydroisoquinoline- 3-carboxamide, a key intermediate for the synthesis of Nelfinavir and Saquinavir, two of the most clinically efficacious anti-AIDS drugs, we attempted to prepare (*S*)-2-benzyl-*N*-*tert*-butyl-1,2,3,4-tetrahydroisoquinoline- 3-carboxamide from (*S*)-2-(benzylamino)-*N*-*tert*-butyl-3 -phenylpropanamide and dimethoxymethane. During this experiment, the title compound, (I), was isolated unexpectedly.

The two planes of phenyl rings make a dihedral angle of 84.1 (1)° (Fig. 1). The absolute configuration (S) of the stereocentre C5 remains unchanged during the synthetic procedure. An X-ray crystal structure determination of the molecular structure of compound (I) was carried out to determine its conformation. The bond lengths are within normal ranges (Allen *et al.*, 1987).

The packing is shown in Fig. 2. The occurrence of weak C—H···O hydrogen bond interactions leads to the formation of linear chains parallel to the *b* axis. The packing is further stabilized by C—H···π interactions (Fig. 2)  with typical geometry (Pavel *et al.*, 1993).

### Experimental

The title compound was prepared by a method based on one described by Jin *et al.* (2005). To a solution of (*S*)-2-(benzylamino)-*N*-*tert*-butyl- 3-phenylpropanamide (11.8 g, 38.1 mmol) in dichloromethane (400 ml) was added dropwise boron trifluoride etherate (13.5 ml, 79.6 mmol) and dimethoxymethane (6.02 g, 79.1 mmol). The mixture was heated to reflux for 48 h. The reaction was quenched by addition of water (90 ml). The solution was adjusted to pH 8 with a 27% aqueous ammonia solution. The organic layer was separated, and the aqueous phase was extracted with dichloromethane. The combined organic phases were washed with brine and dried over Na_2_SO_4_. After filtration and evaporation of the solvents under reduced pressure, the residue was flash chromatographic purification on silica gel (ethyl acetate / petroleum ether = 1 / 4) yielded the product as a white solid. Single crystals were obtained by slow evaporation of a mixture of petroleum ether / dichloromethane solution.

### Refinement

In the absence of anomalous scatterers, Friedel pairs were merged. The absolute configuration was known from the starting material. The hydrogen atoms were positioned geometrically (C—H = 0.93, 0.98, 0.97 or 0.96Å for phenyl, tertiary, methylene or methyl H atoms respectively) and were included in the refinement in the riding model approximation. The displacement parameters of methyl H atoms were set to 1.5*U*_eq_(C), while those of other H atoms were set to 1.2*U*_eq_(C).

### Crystal data

**Table:**

C_21_H_26_N_2_O	*F*_000_ = 696
*M**_r_* = 322.44	*D*_x_ = 1.163 Mg m^−^^3^
Orthorhombic, *P*2_1_2_1_2_1_	Mo *K*α radiation λ = 0.71073 Å
Hall symbol: P 2ac 2ab	Cell parameters from 5367 reflections
*a* = 9.4112 (6) Å	θ = 2.8–32.4º
*b* = 11.4713 (7) Å	µ = 0.07 mm^−^^1^
*c* = 17.0556 (11) Å	*T* = 173 (2) K
*V* = 1841.3 (2) Å^3^	Block, colorless
*Z* = 4	0.62 × 0.45 × 0.23 mm

### Data collection

**Table:**

Bruker APEX CCD diffractometer	2047 independent reflections
Radiation source: fine-focus sealed tube	1824 reflections with *I* > 2σ(*I*)
Monochromator: graphite	*R*_int_ = 0.023
Detector resolution: 16.1903 pixels mm^-1^	θ_max_ = 26.0º
*T* = 173(2) K	θ_min_ = 2.8º
φ and ω scans	*h* = −11→11
Absorption correction: multi-scan(SADABS; Bruker, 2001)	*k* = −11→14
*T*_min_ = 0.957, *T*_max_ = 0.984	*l* = −20→21
8034 measured reflections	

### Refinement

**Table:**

Refinement on *F*^2^	Secondary atom site location: difference Fourier map
Least-squares matrix: full	Hydrogen site location: inferred from neighbouring sites
*R*[*F*^2^ > 2σ(*F*^2^)] = 0.042	H-atom parameters constrained
*wR*(*F*^2^) = 0.107	*w* = 1/[σ^2^(*F*_o_^2^) + (0.0673*P*)^2^ + 0.3582*P*] where *P* = (*F*_o_^2^ + 2*F*_c_^2^)/3
*S* = 1.00	(Δ/σ)_max_ < 0.001
2047 reflections	Δρ_max_ = 0.34 e Å^−^^3^
217 parameters	Δρ_min_ = −0.18 e Å^−^^3^
Primary atom site location: structure-invariant direct methods	Extinction correction: none

### Special details

**Table:**

Geometry. All e.s.d.'s (except the e.s.d. in the dihedral angle between two l.s. planes) are estimated using the full covariance matrix. The cell e.s.d.'s are taken into account individually in the estimation of e.s.d.'s in distances, angles and torsion angles; correlations between e.s.d.'s in cell parameters are only used when they are defined by crystal symmetry. An approximate (isotropic) treatment of cell e.s.d.'s is used for estimating e.s.d.'s involving l.s. planes.
Refinement. Refinement of *F*^2^ against ALL reflections. The weighted *R*-factor *wR* and goodness of fit *S* are based on *F*^2^, conventional *R*-factors *R* are based on *F*, with *F* set to zero for negative *F*^2^. The threshold expression of *F*^2^ > σ(*F*^2^) is used only for calculating *R*-factors(gt) *etc*. and is not relevant to the choice of reflections for refinement. *R*-factors based on *F*^2^ are statistically about twice as large as those based on *F*, and *R*- factors based on ALL data will be even larger.

### Fractional atomic coordinates and isotropic or equivalent isotropic displacement parameters (Å^2^)

**Table:**

	*x*	*y*	*z*	*U*_iso_*/*U*_eq_	
N1	0.3052 (2)	0.09221 (18)	0.72137 (12)	0.0250 (5)	
C2	0.3096 (3)	0.0518 (2)	0.80219 (15)	0.0286 (6)	
H2A	0.3427	−0.0300	0.8054	0.034*	
H2B	0.2152	0.0581	0.8274	0.034*	
N3	0.4114 (2)	0.1312 (2)	0.83816 (13)	0.0310 (5)	
O4	0.6042 (2)	0.2352 (2)	0.79357 (14)	0.0554 (7)	
C4	0.4998 (3)	0.1736 (2)	0.78380 (18)	0.0343 (6)	
C5	0.4499 (3)	0.1293 (2)	0.70449 (16)	0.0296 (6)	
H5	0.5077	0.0596	0.6896	0.036*	
C6	0.2517 (3)	0.0043 (2)	0.66686 (16)	0.0302 (6)	
H6A	0.3094	−0.0674	0.6719	0.036*	
H6B	0.2620	0.0334	0.6125	0.036*	
C7	0.0978 (3)	−0.0245 (2)	0.68211 (14)	0.0273 (6)	
C8	0.0523 (3)	−0.1392 (2)	0.68251 (17)	0.0337 (6)	
H8	0.1188	−0.2004	0.6745	0.040*	
C9	−0.0903 (3)	−0.1651 (3)	0.69453 (19)	0.0424 (7)	
H9	−0.1208	−0.2441	0.6940	0.051*	
C10	−0.1867 (3)	−0.0786 (3)	0.7070 (2)	0.0443 (8)	
H10	−0.2840	−0.0971	0.7153	0.053*	
C11	−0.1422 (3)	0.0367 (3)	0.7075 (2)	0.0458 (8)	
H11	−0.2087	0.0975	0.7166	0.055*	
C12	−0.0008 (3)	0.0628 (2)	0.69456 (18)	0.0362 (7)	
H12	0.0291	0.1419	0.6942	0.043*	
C13	0.4632 (4)	0.2208 (3)	0.64056 (17)	0.0399 (7)	
H13A	0.3822	0.2753	0.6457	0.048*	
H13B	0.5510	0.2660	0.6504	0.048*	
C14	0.4673 (3)	0.1777 (2)	0.55702 (17)	0.0326 (6)	
C15	0.3770 (4)	0.2246 (3)	0.5011 (2)	0.0469 (8)	
H15	0.3086	0.2813	0.5161	0.056*	
C16	0.3856 (4)	0.1894 (3)	0.4231 (2)	0.0553 (10)	
H16	0.3238	0.2229	0.3853	0.066*	
C17	0.4815 (4)	0.1078 (3)	0.40063 (19)	0.0508 (9)	
H17	0.4882	0.0852	0.3472	0.061*	
C18	0.5691 (3)	0.0578 (3)	0.45596 (19)	0.0476 (8)	
H18	0.6343	−0.0013	0.4409	0.057*	
C19	0.5622 (3)	0.0934 (3)	0.53324 (18)	0.0399 (7)	
H19	0.6242	0.0591	0.5707	0.048*	
C20	0.4334 (3)	0.1408 (3)	0.92442 (17)	0.0382 (7)	
C21	0.5676 (6)	0.0773 (5)	0.9454 (3)	0.0904 (15)	
H21A	0.5591	−0.0048	0.9301	0.109*	
H21B	0.5835	0.0825	1.0020	0.109*	
H21C	0.6478	0.1127	0.9176	0.109*	
C22	0.3043 (5)	0.0931 (5)	0.9661 (2)	0.0855 (14)	
H22A	0.2931	0.0102	0.9535	0.103*	
H22B	0.2197	0.1359	0.9490	0.103*	
H22C	0.3163	0.1022	1.0229	0.103*	
C23	0.4439 (5)	0.2670 (3)	0.9470 (2)	0.0604 (10)	
H23A	0.5271	0.3019	0.9216	0.091*	
H23B	0.4536	0.2735	1.0040	0.091*	
H23C	0.3579	0.3081	0.9300	0.091*	

### Atomic displacement parameters (Å^2^)

**Table:**

	*U*^11^	*U*^22^	*U*^33^	*U*^12^	*U*^13^	*U*^23^
N1	0.0249 (10)	0.0266 (10)	0.0235 (11)	−0.0031 (9)	0.0000 (9)	−0.0017 (9)
C2	0.0307 (13)	0.0274 (12)	0.0277 (13)	−0.0071 (11)	−0.0031 (11)	0.0001 (11)
N3	0.0279 (11)	0.0358 (12)	0.0293 (12)	−0.0077 (10)	−0.0043 (9)	−0.0024 (10)
O4	0.0406 (12)	0.0721 (16)	0.0535 (14)	−0.0296 (12)	0.0094 (11)	−0.0233 (12)
C4	0.0277 (12)	0.0354 (14)	0.0397 (15)	−0.0057 (12)	0.0037 (13)	−0.0089 (12)
C5	0.0281 (13)	0.0283 (12)	0.0324 (14)	−0.0034 (11)	0.0060 (12)	−0.0057 (11)
C6	0.0331 (13)	0.0300 (13)	0.0275 (13)	−0.0049 (12)	0.0008 (11)	−0.0073 (11)
C7	0.0325 (14)	0.0295 (12)	0.0198 (11)	−0.0056 (11)	−0.0042 (11)	0.0003 (10)
C8	0.0406 (15)	0.0293 (13)	0.0314 (14)	−0.0049 (12)	−0.0028 (12)	0.0004 (12)
C9	0.0476 (17)	0.0374 (15)	0.0422 (17)	−0.0168 (14)	−0.0120 (14)	0.0079 (13)
C10	0.0304 (14)	0.0547 (19)	0.0479 (18)	−0.0117 (14)	−0.0080 (14)	0.0090 (15)
C11	0.0316 (15)	0.0472 (18)	0.059 (2)	0.0009 (13)	−0.0099 (15)	0.0036 (16)
C12	0.0336 (14)	0.0299 (13)	0.0450 (16)	−0.0025 (12)	−0.0067 (13)	−0.0008 (13)
C13	0.0526 (18)	0.0293 (13)	0.0378 (17)	−0.0078 (14)	0.0140 (14)	−0.0011 (12)
C14	0.0351 (14)	0.0285 (13)	0.0342 (15)	−0.0089 (12)	0.0063 (12)	0.0038 (11)
C15	0.0500 (19)	0.0334 (16)	0.057 (2)	0.0005 (15)	−0.0022 (16)	0.0135 (16)
C16	0.059 (2)	0.060 (2)	0.047 (2)	−0.0115 (19)	−0.0159 (18)	0.0226 (17)
C17	0.052 (2)	0.067 (2)	0.0334 (16)	−0.0305 (19)	0.0027 (15)	0.0044 (15)
C18	0.0399 (17)	0.061 (2)	0.0414 (17)	−0.0065 (16)	0.0117 (15)	−0.0079 (16)
C19	0.0341 (15)	0.0501 (17)	0.0355 (16)	−0.0011 (14)	0.0020 (13)	0.0011 (14)
C20	0.0396 (15)	0.0439 (17)	0.0311 (15)	−0.0076 (14)	−0.0108 (12)	0.0003 (13)
C21	0.103 (3)	0.095 (3)	0.073 (3)	0.036 (3)	−0.038 (2)	−0.012 (2)
C22	0.098 (3)	0.120 (3)	0.0391 (18)	−0.052 (3)	−0.003 (2)	−0.001 (2)
C23	0.084 (3)	0.058 (2)	0.0385 (19)	−0.011 (2)	0.003 (2)	−0.0113 (16)

### Geometric parameters (Å, °)

**Table:**

N1—C2	1.455 (3)	C13—C14	1.508 (4)
N1—C5	1.455 (3)	C13—H13A	0.9900
N1—C6	1.461 (3)	C13—H13B	0.9900
C2—N3	1.457 (3)	C14—C19	1.378 (4)
C2—H2A	0.9900	C14—C15	1.387 (4)
C2—H2B	0.9900	C15—C16	1.392 (5)
N3—C4	1.336 (4)	C15—H15	0.9500
N3—C20	1.490 (4)	C16—C17	1.355 (5)
O4—C4	1.222 (3)	C16—H16	0.9500
C4—C5	1.519 (4)	C17—C18	1.378 (5)
C5—C13	1.519 (4)	C17—H17	0.9500
C5—H5	1.0000	C18—C19	1.381 (5)
C6—C7	1.508 (4)	C18—H18	0.9500
C6—H6A	0.9900	C19—H19	0.9500
C6—H6B	0.9900	C20—C21	1.501 (5)
C7—C12	1.381 (4)	C20—C23	1.502 (5)
C7—C8	1.384 (4)	C20—C22	1.510 (5)
C8—C9	1.390 (4)	C21—H21A	0.9800
C8—H8	0.9500	C21—H21B	0.9800
C9—C10	1.362 (4)	C21—H21C	0.9800
C9—H9	0.9500	C22—H22A	0.9800
C10—C11	1.388 (5)	C22—H22B	0.9800
C10—H10	0.9500	C22—H22C	0.9800
C11—C12	1.382 (4)	C23—H23A	0.9800
C11—H11	0.9500	C23—H23B	0.9800
C12—H12	0.9500	C23—H23C	0.9800
			
C2—N1—C5	104.7 (2)	C5—C13—H13A	108.0
C2—N1—C6	113.1 (2)	C14—C13—H13B	108.0
C5—N1—C6	113.5 (2)	C5—C13—H13B	108.0
N1—C2—N3	102.62 (19)	H13A—C13—H13B	107.3
N1—C2—H2A	111.2	C19—C14—C15	117.9 (3)
N3—C2—H2A	111.2	C19—C14—C13	121.6 (3)
N1—C2—H2B	111.2	C15—C14—C13	120.5 (3)
N3—C2—H2B	111.2	C14—C15—C16	120.6 (3)
H2A—C2—H2B	109.2	C14—C15—H15	119.7
C4—N3—C2	110.1 (2)	C16—C15—H15	119.7
C4—N3—C20	124.9 (2)	C17—C16—C15	120.7 (3)
C2—N3—C20	123.6 (2)	C17—C16—H16	119.7
O4—C4—N3	128.0 (3)	C15—C16—H16	119.7
O4—C4—C5	124.3 (3)	C16—C17—C18	119.4 (3)
N3—C4—C5	107.7 (2)	C16—C17—H17	120.3
N1—C5—C13	114.9 (2)	C18—C17—H17	120.3
N1—C5—C4	102.2 (2)	C17—C18—C19	120.2 (3)
C13—C5—C4	112.5 (2)	C17—C18—H18	119.9
N1—C5—H5	109.0	C19—C18—H18	119.9
C13—C5—H5	109.0	C14—C19—C18	121.2 (3)
C4—C5—H5	109.0	C14—C19—H19	119.4
N1—C6—C7	111.9 (2)	C18—C19—H19	119.4
N1—C6—H6A	109.2	N3—C20—C21	108.4 (3)
C7—C6—H6A	109.2	N3—C20—C23	109.5 (3)
N1—C6—H6B	109.2	C21—C20—C23	110.6 (3)
C7—C6—H6B	109.2	N3—C20—C22	109.1 (3)
H6A—C6—H6B	107.9	C21—C20—C22	112.9 (4)
C12—C7—C8	118.7 (3)	C23—C20—C22	106.4 (3)
C12—C7—C6	120.9 (2)	C20—C21—H21A	109.5
C8—C7—C6	120.4 (3)	C20—C21—H21B	109.5
C7—C8—C9	120.2 (3)	H21A—C21—H21B	109.5
C7—C8—H8	119.9	C20—C21—H21C	109.4
C9—C8—H8	119.9	H21A—C21—H21C	109.5
C10—C9—C8	120.7 (3)	H21B—C21—H21C	109.5
C10—C9—H9	119.6	C20—C22—H22A	109.5
C8—C9—H9	119.6	C20—C22—H22B	109.4
C9—C10—C11	119.6 (3)	H22A—C22—H22B	109.5
C9—C10—H10	120.2	C20—C22—H22C	109.5
C11—C10—H10	120.2	H22A—C22—H22C	109.5
C12—C11—C10	119.8 (3)	H22B—C22—H22C	109.5
C12—C11—H11	120.1	C20—C23—H23A	109.5
C10—C11—H11	120.1	C20—C23—H23B	109.5
C7—C12—C11	121.0 (3)	H23A—C23—H23B	109.5
C7—C12—H12	119.5	C20—C23—H23C	109.5
C11—C12—H12	119.5	H23A—C23—H23C	109.5
C14—C13—C5	117.0 (2)	H23B—C23—H23C	109.5
C14—C13—H13A	108.0		
			
C5—N1—C2—N3	35.3 (3)	C8—C9—C10—C11	0.2 (5)
C6—N1—C2—N3	159.4 (2)	C9—C10—C11—C12	0.6 (5)
N1—C2—N3—C4	−25.0 (3)	C8—C7—C12—C11	0.2 (4)
N1—C2—N3—C20	168.0 (2)	C6—C7—C12—C11	179.1 (3)
C2—N3—C4—O4	−174.1 (3)	C10—C11—C12—C7	−0.8 (5)
C20—N3—C4—O4	−7.3 (5)	N1—C5—C13—C14	−84.6 (3)
C2—N3—C4—C5	4.8 (3)	C4—C5—C13—C14	159.1 (3)
C20—N3—C4—C5	171.5 (3)	C5—C13—C14—C19	−52.8 (4)
C2—N1—C5—C13	−154.4 (2)	C5—C13—C14—C15	128.8 (3)
C6—N1—C5—C13	81.8 (3)	C19—C14—C15—C16	−1.8 (5)
C2—N1—C5—C4	−32.3 (3)	C13—C14—C15—C16	176.6 (3)
C6—N1—C5—C4	−156.1 (2)	C14—C15—C16—C17	0.7 (5)
O4—C4—C5—N1	−163.8 (3)	C15—C16—C17—C18	1.2 (5)
N3—C4—C5—N1	17.3 (3)	C16—C17—C18—C19	−2.0 (5)
O4—C4—C5—C13	−40.1 (4)	C15—C14—C19—C18	1.0 (4)
N3—C4—C5—C13	141.0 (2)	C13—C14—C19—C18	−177.4 (3)
C2—N1—C6—C7	65.6 (3)	C17—C18—C19—C14	0.9 (5)
C5—N1—C6—C7	−175.3 (2)	C4—N3—C20—C21	−62.2 (4)
N1—C6—C7—C12	45.6 (3)	C2—N3—C20—C21	102.8 (4)
N1—C6—C7—C8	−135.5 (3)	C4—N3—C20—C23	58.5 (4)
C12—C7—C8—C9	0.6 (4)	C2—N3—C20—C23	−136.5 (3)
C6—C7—C8—C9	−178.3 (3)	C4—N3—C20—C22	174.5 (3)
C7—C8—C9—C10	−0.8 (5)	C2—N3—C20—C22	−20.5 (4)

### Hydrogen-bond geometry (Å, °)

**Table:**

*D*—H···*A*	*D*—H	H···*A*	*D*···*A*	*D*—H···*A*
C6—H6A···O4^i^	0.99	2.48	3.439 (4)	164
C17—H17···Cg^ii^	0.95	2.68	3.621 (4)	169

Symmetry codes: (i) −*x*+1, *y*−1/2, −*z*+3/2; (ii) −*x*+1/2, −*y*, *z*−1/2.
